# The Effects of *Rhizophagus irregularis* Inoculation on Transcriptome of *Medicago lupulina* Leaves at Early Vegetative and Flowering Stages of Plant Development

**DOI:** 10.3390/plants12203580

**Published:** 2023-10-15

**Authors:** Andrey P. Yurkov, Alexey M. Afonin, Alexey A. Kryukov, Anastasia O. Gorbunova, Tatyana R. Kudryashova, Anastasia I. Kovalchuk, Anastasia I. Gorenkova, Ekaterina M. Bogdanova, Yuri V. Kosulnikov, Yuri V. Laktionov, Andrey P. Kozhemyakov, Daria A. Romanyuk, Vladimir A. Zhukov, Roman K. Puzanskiy, Yulia V. Mikhailova, Vladislav V. Yemelyanov, Maria F. Shishova

**Affiliations:** 1 Laboratory of Ecology of Symbiotic and Associative Rhizobacteria, All-Russia Research Institute for Agricultural Microbiology, Pushkin, St. Petersburg 196608, Russia; afoninalexeym@gmail.com (A.M.A.); rainniar13@gmail.com (A.A.K.); gorbunova.anastasia93@mail.ru (A.O.G.); tahacorfu@yandex.ru (T.R.K.); k.nastya4321@gmail.com (A.I.K.); nastya.gorenkova.2016@mail.ru (A.I.G.); bogdanova.ekaterina15@gmail.com (E.M.B.); kullavayn@gmail.com (Y.V.K.); laktionov@list.ru (Y.V.L.); kojemyakov@rambler.ru (A.P.K.); 2 Graduate School of Biotechnology and Food Science, Peter the Great St. Petersburg Polytechnic University, St. Petersburg 194064, Russia; 3 Faculty of Biology, St. Petersburg State University, St. Petersburg 199034, Russia; puzansky@yandex.ru (R.K.P.); bootika@mail.ru (V.V.Y.); mshishova@mail.ru (M.F.S.); 4 Laboratory of Genetics of Plant-Microbe Interactions, All-Russia Research Institute for Agricultural Microbiology, Pushkin, St. Petersburg 196608, Russia; d.romanyuk@arriam.ru (D.A.R.); vzhukov@arriam.ru (V.A.Z.); 5 Laboratory of Analytical Phytochemistry, Komarov Botanical Institute of the Russian Academy of Sciences, St. Petersburg 197022, Russia; 6 Laboratory of Biosystematics and Cytology, Komarov Botanical Institute of the Russian Academy of Sciences, St. Petersburg 197022, Russia; ymikhaylova@binran.ru

**Keywords:** *Medicago lupulina*, arbuscular mycorrhiza, *Rhizophagus irregularis*, symbiotic efficiency, mycorrhizal growth response, plant development, physiological stage, leaf, transcriptome, phosphorus deficiency

## Abstract

The study is aimed at revealing the effects of *Rhizophagus irregularis* inoculation on the transcriptome of *Medicago lupulina* leaves at the early (second leaf formation) and later (flowering) stages of plant development. A pot experiment was conducted under conditions of low phosphorus (P) level in the substrate. *M. lupulina* plants were characterized by high mycorrhizal growth response and mycorrhization parameters. Library sequencing was performed on the Illumina HiseqXTen platform. Significant changes in the expression of 4863 (*p_adj_* < 0.01) genes from 34049 functionally annotated genes were shown by Massive Analysis of cDNA Ends (MACE-Seq). GO enrichment analysis using the Kolmogorov–Smirnov test was performed, and 244 functional GO groups were identified, including genes contributing to the development of effective AM symbiosis. The Mercator online tool was used to assign functional classes of differentially expressed genes (DEGs). The early stage was characterized by the presence of six functional classes that included only upregulated GO groups, such as genes of carbohydrate metabolism, cellular respiration, nutrient uptake, photosynthesis, protein biosynthesis, and solute transport. At the later stage (flowering), the number of stimulated GO groups was reduced to photosynthesis and protein biosynthesis. All DEGs of the GO:0016036 group were downregulated because AM plants had higher resistance to phosphate starvation. For the first time, the upregulation of genes encoding thioredoxin in AM plant leaves was shown. It was supposed to reduce ROS level and thus, consequently, enhance the mechanisms of antioxidant protection in *M. lupulina* plants under conditions of low phosphorus level. Taken together, the obtained results indicate genes that are the most important for the effective symbiosis with *M. lupulina* and might be engaged in other plant species.

## 1. Introduction

One of the pressing issues in biology involves discerning the mechanisms that govern the formation and development of plant–microbial systems (PMS) integrating arbuscular mycorrhizal (AM) fungi. The list of possible effectors is still growing. For example, recently, about a hundred symbiosin genes specific to microbial–plant interactions have been added [1]. Mechanisms of plant growth enhancement under AM fungi inoculation, i.e., mechanisms of symbiotic efficiency (“Mycorrhizal Growth Response”, MGR [2]), are the most debated ones despite a huge number of investigations. There are several reasons for that. In general, high MGR is not observed in the models used for such studies. Another one is the complexity of result comparison due to discrepancies in the development stages for different plant species, even if they are harvested on the same day after inoculation (DAI). In addition, investigations of PMS are carried out on a wide spectrum of plant species (>30), but only a few species of AM fungi are analyzed in model experiments due to their nonspecific interaction: mainly *Rhizophagus irregularis*, and less so *Funneliformis mosseae*, *Gigaspora margarita*, etc. [3]. At the same time, plants often have low MGR under standard conditions [2,4,5,6,7]. Lines with higher MGR were found among the following plant species: four *Sorghum bicolor* lines under *R. irregularis* interaction with MGR (*p* < 0.05) ~50–200% [6], and the J5 *Medicago truncatula* var Jemalong line with MGR > 150% [8]. The second factor that may be crucial for the interpretation of obtained results is associated with non-standardized time points (DAI) of data collection, which often do not coincide with days after sowing [4,7,9,10,11,12,13,14,15,16,17,18]. Thus, in different PMS models and at different stages of plant development, too diverse data on metabolites, gene expression, and protein composition were obtained due to the high variation in the responses of symbiosis partners from mutualism to parasitism. These data are difficult to interpret separately and compare with each other.

Therefore, generally, the mechanisms controlling the development of an efficient AM have not yet been determined. The selection of PMS with high AM efficiency, unification of examined time points according to beginning of each new host plant development stage, and application of complex methods of their research using approaches of systems biology will contribute to solving the problem. In recent years, the number of studies using proteomics and metabolomics has been increasing. The advent of *omics* technologies has provided the possibility of quick and effective means of filling in the gaps related to difficulties in comparing data obtained at different developmental stages for different plant species. Special interest is always paid to the improvement of sequencing and transcriptome analyses [19,20,21,22,23]. Studies are underway investigating the influence of AM fungi on the proteome of *M. truncatula*, *Pisum sativum* [23,24,25,26,27], and on the metabolome (with gaschromatography—mass spectrometry, GC-MS) of *M. truncatula*, *M. lupulina*, *P. sativum* [26,28,29,30,31,32], etc. The role of AM transcriptomic studies of *M. truncatula*, *Nicotiana attenuata*, *P. sativum* is remarkable [33,34,35]. Among other *omics* technologies, transcriptomic analysis stands as one of the most crucial ones in studying the formation and functions of AM symbiosis at the molecular level. The ability to capture “snapshots” of the symbiotic system at various stages of the symbiosis allows not only for determining the genes involved in the development of both micro- and macro-symbionts, but also makes it possible to determine the external factors affecting the system and the direction of tissue development at any given time. Recently, next-generation sequencing (NGS) methods such as RNA-Seq and multiple-genome coverage sequencing have been actively developed [36]. A promising modification of RNA-Seq is the Massive Analysis of cDNA Ends (MACE-Seq), which generates only one sequence from the 3′-end of each polyadenylated transcript [37]. Since sequencing reads are concentrated on a small part of the transcript, a relatively small number of sequences is usually sufficient to reliably cover polymorphisms in sparsely expressed sequences [38]. MACE-Seq has been successfully applied in parasitic PMS for genetic mapping of the stem rust resistance locus in perennial *Lolium perenne* [39], and for anthracnose resistance in *Lupinus angustifolius* [40]. The first use of MACE-Seq for the analysis of AM symbiosis genes was conducted in 2021 [41]. It has been shown that in the roots of *P. sativum* lines with high AM efficiency, a higher transcription level of some genes (upregulated genes) was observed: the gene encoding the contact site protein VAP27 (probably participating in membrane structure development during arbuscule formation), the gene encoding phosphocholine phosphatase (probably involved in lipid metabolism, which is necessary for AM function [42,43]), and four genes involved in flavonoid biosynthesis. However, it should be noted that in this work, analyzed efficient *P. sativum* lines were selected not by MGR (upon mono-inoculation with a single AM fungal strain), but by EIBSM (“Effectiveness of Interaction with Beneficial Soil Microbes” [44]), i.e., upon treatment with a complex microbial fertilizer containing both AM fungi and bacteria. However, the EIBSM calculated on the basis of plant dry weight for AM-effective *P. sativum* lines was not high, at ~25–50% [45]. Thus, transcriptome analysis of obligately mycotrophic lines with an extremely high response to mono-inoculation with AM fungal strain (according to MGR) has not yet been conducted. The transcriptomic analysis of the model PMS “*Medicago lupulina* + *Rhizophagus irregularis*”, with MGR > 350% under conditions of phosphorus (P) deficiency for plant nutrition is promising [46]. The micro-vegetative experiments in *M. lupulina* provided in our earlier experiments during the development of AM symbiosis showed significant changes in the course of development and hormonal rearrangements in the host plant [47,48]; metabolic rearrangements [30,49]; and changes in the expression of a number of genes for carbohydrate metabolism and P transport [50].

Considering the above, the aim of this research was to evaluate the influence of inoculation with effective AM fungus (*Rhizophagus irregularis*) on the transcription profiles in leaves of the highly responsive obligate mycotrophic *Medicago lupulina* MlS-1 line during the development of the host plant from the early vegetative stage (second leaf development) to the later flowering stage under conditions of P deficiency.

## 2. Results

### 2.1. The Effect of AM Fungus on Medicago Lupulina at Second Leaf and Flowering Stages of Plant Development

**Figure 1:**
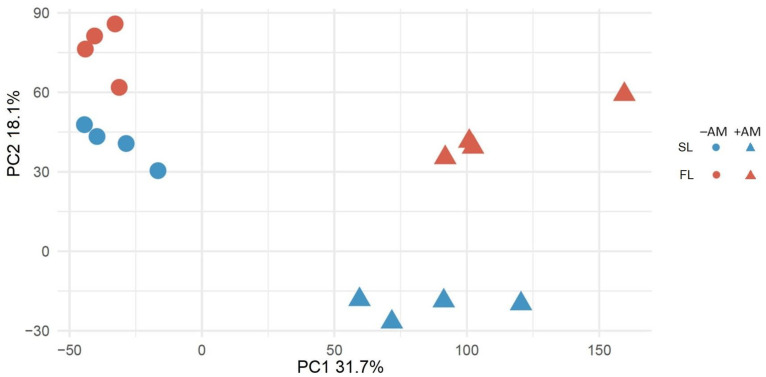
PCA score plot for unsupervised analysis of leaf transcriptome profiles of mycorrhized (+AM) and nonmycorrhized (−AM) *Medicago lupulina* MlS-1 plants sampled at 24th (second leaf development – “SL”) and 48th (flowering – “FL”) day after sowing and inoculation.

The data obtained indicate that the *M. lupulina* MlS-1 line was characterized by a significant MGR under inoculation with *R. irregularis* fungus, both by the fresh weight of the aboveground parts and roots, both at early and late stages of plant development (Figure S1A). This points to a substantial AM efficiency of model PMS under conditions of low Pi in substrate with noticeable development of mycelium and arbuscules of AM fungus in *M. lupulina* roots (Figure S1B,C). The formation and development of an effective symbiosis caused serious genetic rearrangements in the plant. MACE sequences of 34,049 genes are presented in the NCBI database as PRJNA873716 BioProject with SAMN30499749-SAMN30499752 biosamples. Principal Component Analysis (PCA) of *M. lupulina* MlS-1 leaf transcriptome profiles showed that the transcription of these genes differs intensively in the “+AM” and “−AM” variants, as well as depending on the host plant development stage, “Second leaf” (SL) and “Flowering” (FL) (Figure 1).

**Figure 2:**
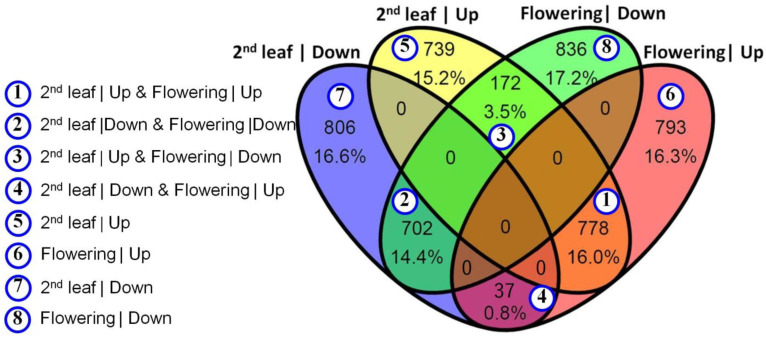
Venn diagram for *M. lupulina* genes characterized by alterations in the expression level under the influence of mycorrhization with *R. irregularis*. The expression was estimated for +AM plants at “Second leaf” and “Flowering” stages relative to the expression for −AM control plants at the corresponding stages (*p_adj_* < 0.01). The regulation of different groups of DEGs is marked with numbers in circles: (1) “① 2nd leaf|Up & Flowering|Up”, upregulation at “Second leaf” and “Flowering” stages; (2) “② 2nd leaf|Down & Flowering|Down”, downregulation at both stages; (3) “③ 2nd leaf|Up & Flowering|Down”, shift of upregulation at “Second leaf” stage to downregulation at “Flowering” stage; (4) “④ 2nd leaf|Down & Flowering|Up”, shift of downregulation at “Second leaf” stage to upregulation at “Flowering” stage; (5) “⑤ 2nd leaf|Up”, upregulation at “Second leaf” stage; (6) “⑥ Flowering|Up”, upregulation at “Flowering” stage; (7) “⑦ 2nd leaf|Down”, downregulation at “Second leaf” stage; (8) “⑧ Flowering|Down”, downregulation at “Flowering” stage.

The intersection of gene groups differs in the expression level between the *M. lupulina* MlS-1 line plants with AM and without AM under conditions of low P levels in the substrate (Figure 2). It confirms the shift in gene expression resulting from the inoculation and the host plant developmental stage. The number of genes with a multidirectional effect of AM on their expression at SL and FL stages of plant development (at early and later stages of AM symbiosis; “172 upregulated at SL, but downregulated at FL” + “37 downregulated at SL, but upregulated at FL” = 209 genes) is almost an order of magnitude less than the genes that respond equally in terms of expression to the presence of AM fungus (“778 upregulated at SL and FL” + “702 downregulated at SL and FL” = 1480 genes). Zero values in seven groups out of fourteen groups are due to the assumption that the same gene cannot have up- and downregulations at the same time (Figure 2). The obtained data indicate a facility for determining a number of functional groups of genes involved in the development of effective AM symbiosis.

### 2.2. Differential Gene Expression Determined by the Interaction of Medicago Lupulina with Rhizophagus irregularis

**Figure 3:**
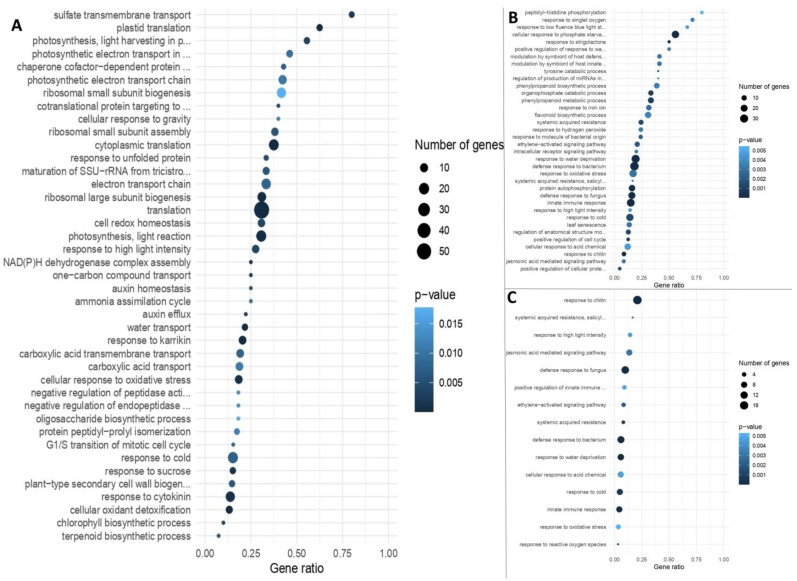

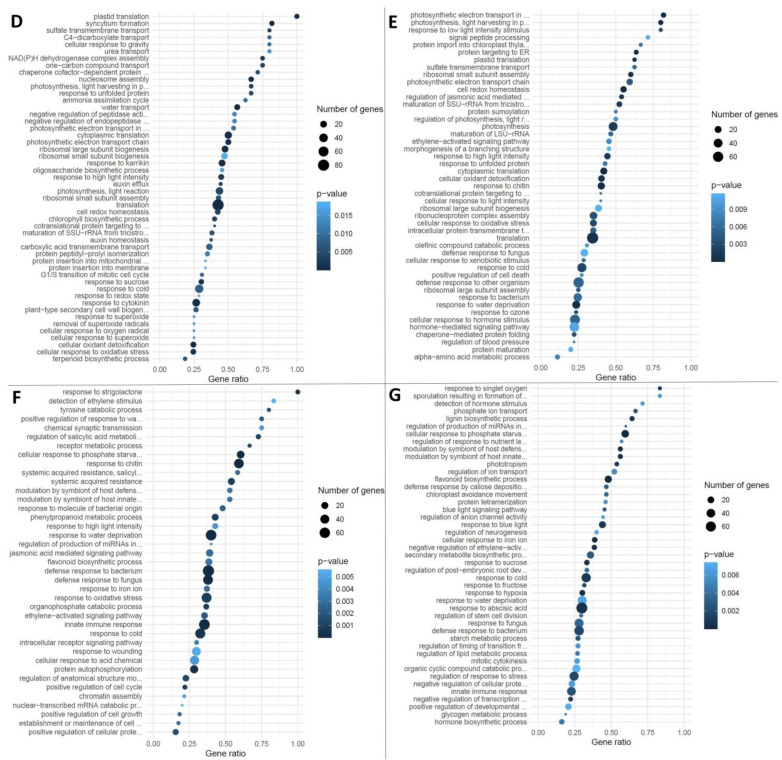
GO groups: Biological process for leaf *M. lupulina* genes with different regulations. (**A**) Upregulation at both 2nd leaf formation and flowering stage under conditions of inoculation with *R. irregularis* (see ① in Figure 2). (**B**) Downregulation at both 2nd leaf formation and flowering stage under conditions of inoculation with *R. irregularis* (see ② in Figure 2). (**C**) Upregulation at 2nd leaf formation and downregulation at flowering stage under conditions of inoculation with *R. irregularis* (see ③ in Figure 2). (**D**) Upregulation at 2nd leaf formation stage under conditions of inoculation with *R. irregularis* (see ⑤ in Figure 2). (**E**) upregulation at flowering stage under conditions of inoculation with *R. irregularis* (see ⑥ in Figure 2). (**F**) Downregulation at 2nd leaf formation stage under conditions of inoculation with *R. irregularis* (see ⑦ in Figure 2). (**G**) Downregulation at flowering stage under conditions of inoculation with *R. irregularis* (see ⑧ in Figure 2). Abbreviated names of biological processes: cellular response to phosphate starvation; chaperone cofactor-dependent protein refolding; cotranslational protein targeting to membrane; defense response by callose deposition in cell wall; establishment or maintenance of cell polarity; intracellular protein transmembrane transport; maturation of SSU-rRNA from tricistronic rRNA transcript (SSU-rRNA, 5.8S rRNA, LSU-rRNA); modulation by symbiont of host defense response; modulation by symbiont of host innate immune response; negative regulation of cellular protein metabolic process; negative regulation of endopeptidase activity; negative regulation of ethylene-activated signaling pathway; negative regulation of peptidase activity; negative regulation of transcription by RNA polymerase II; nuclear-transcribed mRNA catabolic process, nonsense-mediated decay; organic cyclic compound catabolic process; photosynthesis, light harvesting in photosystem I; photosynthetic electron transport chain; photosynthetic electron transport in photosystem I; plant-type secondary cell wall biogenesis; positive regulation of cellular protein metabolic process; positive regulation of developmental process; positive regulation of innate immune response; positive regulation of response to water deprivation; protein import into chloroplast thylakoid membrane; protein insertion into mitochondrial membrane; regulation of anatomical structure morphogenesis; regulation of jasmonic acid-mediated signaling pathway; regulation of photosynthesis, light reaction; regulation of post-embryonic root development; regulation of production of miRNAs involved in gene silencing by miRNA; regulation of response to nutrient levels; regulation of salicylic acid metabolic process; regulation of timing of transition from vegetative to reproductive phase; secondary metabolite biosynthetic process; systemic acquired resistance, salicylic acid-mediated signaling pathway; chaperone cofactor-dependent protein refolding; photosynthesis, light harvesting in photosystem I; photosynthetic electron transport in photosystem I; positive regulation of response to water deprivation; response to low-fluence blue light stimulus by blue low-fluence system; sporulation resulting in formation of cellular spore; systemic acquired resistance, salicylic acid-mediated signaling pathway. GO groups: Biological process for leaf *M. lupulina* genes with different regulations.

Sequenced reads were compared with the reference genome of *M. truncatula* presented in BioProjects PRJNA702529, total length (Mb): 430,008, protein count: 42,683, GC%: 33.4385 [51,52]. As a result of the GO analysis, 4863 differentially expressed genes (DEGs) were identified. It allowed for the reveal of the enrichment of three of four groups of genes with intersections in regulation between SL and FL stages of development (Figure 3A–C and Figure S2A–C) and of all four groups without intersections in regulation for SL and FL development stages (Figure 3D–G and Figure S2D–G).

Let us consider in detail the groups with DEG intersections of SL and FL development stages (Figure 2). A total of 41 GO groups were identified for DEGs (up to 58 DEGs in one GO group), characterized by upregulation during mycorrhization at both SL and FL development stages (Figure 3A; “①” in Figure 2). The three most represented groups are detected with a gene ratio close to 0.5 (Table S1). DEGs from various GO groups have been combined into the 12 most common basic functional classes (categories) of biological processes using the Mercator pipeline (Table S1). The only specific GO group for DEGs with upregulation under conditions of inoculation with *R. irregularis* at both early and late stages of *M. lupulina* development was the “carboxylic acid transport” group with a low gene ratio (Figure 3A; Table S1).

The results of our study showed that downregulation under mycorrhization at both stages of host plant development is characteristic of DEGs from 36 GO groups (up to 34 DEGs in one GO group; Figure 3B; “②” in Figure 2). The five most represented groups are detected with a gene ratio >0.5 (Table S1). DEGs have been combined into the seven most common basic functional classes of biological processes (Table S1). Seven specific GO groups for DEGs with downregulation under mycorrhization with *R. irregularis* were identified both at early and later stages of *M. lupulina* development (Figure 3B): peptidyl-histidine phosphorylation; response to low-fluence blue light stimulus by blue low-fluence system; response to strigolactone; phenylpropanoid biosynthetic process; response to hydrogen peroxide; leaf senescence; positive regulation of cellular protein metabolic process.

Upregulation at the second leaf development stage with downregulation at the flowering stage under mycorrhization was noted for DEGs from 15 GO groups (up to 17 DEGs in one GO group; Figure 3C; “③” in Figure 2); groups with a gene ratio >0.5 are absent. The most represented group was “response to chitin” (GO:0010200). DEGs have combined into the three most common main functional classes of biological processes with low group enrichment and low gene ratio (Table S1). Two specific GO groups of DEGs were identified: positive regulation of innate immune response and response to reactive oxygen species. According to the obtained results, 37 genes (see “④” in Figure 2) downregulated at the second leaf development and upregulated at the flowering stage under conditions of inoculation with *R. irregularis* were not grouped into enrichment groups. Consider the GO groups of DEGs that do not have intersections between developmental stages (SL and FL).

Reverse dynamics was revealed for fifty groups of GO for DEGs (up to 80 DEGs in one group of GO) characterized by upregulation under mycorrhization only at the second leaf development stage (without intersections with the flowering stage; Figure 3D; “⑤” in Figure 2). The eight most represented groups are detected with a gene ratio >0.5 (Table S1), including plastid translation with a gene ratio = 1.0 (!). DEGs have been combined into the 12 most common basic functional classes of biological processes (Table S1). A number of specific GO groups for DEGs with upregulation under mycorrhization with *R. irregularis* were identified at the early stage of *M. lupulina* development (Figure 3D), namely C_4_-dicarboxylate transport; water transport; urea transport; response to redox state; and other groups with lower enrichment.

It was noted that 47 GO groups for DEGs (up to 75 DEGs in one GO group) were characterized by upregulation under mycorrhization only at the flowering development stage (without intersections with the second leaf development stage; Figure 3E; “⑥” in Figure 2). The eleven most represented groups are detected with a gene ratio >0.5 (Table S1). DEGs have been combined into the 10 most common basic functional classes of biological processes (Table S1). A significant number of specific GO groups of DEGs have been shown with upregulation under mycorrhization with *R. irregularis* only at the late stage of *M. lupulina* development, at the flowering stage (Figure 3E), namely regulation of photosynthesis, light reaction; photosynthesis; protein import into chloroplast thylakoid membrane; protein targeting to ER; regulation of jasmonic acid-mediated signaling pathway; intracellular protein transmembrane transport; ribonucleoprotein complex assembly; cellular response to xenobiotic stimulus; defense response to other organism; ribosomal large subunit assembly; response to bacterium; cellular response to hormone stimulus; hormone-mediated signaling pathway; alpha-amino acid metabolic process; and other groups with lower enrichment.

In the present study, 39 GO groups of DEGs (up to 71 DEGs in one GO group) characterized by downregulation under mycorrhization only at the second leaf development stage (without intersections with FL stage; Figure 3F; “⑦” in Figure 2) were determined. The eleven most represented groups are detected with a gene ratio >0.5 (Table S1), including response to strigolactone with a gene ratio = 1.0 (!). DEGs have been grouped into the seven most common basic functional classes of biological processes (Table S1). A significant number of specific GO groups of DEGs with downregulation under mycorrhization with *R. irregularis* have been shown only at the early stage of *M. lupulina* development, at the stage of second leaf development (Figure 3F), namely response to strigolactone; regulation of salicylic acid metabolic process; detection of ethylene stimulus; chromatin assembly; positive regulation of cell growth; positive regulation of cellular protein metabolic process; and other groups with lower enrichment.

A total of 45 GO groups of DEGs (up to 73 DEGs in one GO group) were characterized by downregulation under mycorrhization only at the FL stage (without intersections with the beginning of the second leaf development stage; Figure 3G; “⑧” in Figure 2). The nine most represented groups are detected with a gene ratio >0.5 (Table S1). DEGs have been combined into the eight most common basic functional classes of biological processes (Table S1). A significant number of specific GO groups of DEGs with downregulation under mycorrhization with *R. irregularis* have been shown only at the late stage of *M. lupulina* development, at the flowering development stage (Figure 3G), namely detection of hormone stimulus; phosphate ion transport; lignin biosynthetic process; phototropism; regulation of ion transport; defense response by callose deposition in cell wall; chloroplast avoidance movement; regulation of anion channel activity; cellular response to iron ion; secondary metabolite biosynthetic process; negative regulation of ethylene-activated signaling pathway; response to fructose; response to abscisic acid; regulation of stem cell division; response to fungus; starch metabolic process; regulation of timing of transition from vegetative to reproductive phase; regulation of lipid metabolic process; organic cyclic compound catabolic process; regulation of response to stress; negative regulation of cellular protein metabolic process; positive regulation of developmental process; glycogen metabolic process; and other groups with lower enrichment.

The calculation of enrichment groups for GO “Molecular function” groups is presented in Figure S2A–G.

**Figure 4:**
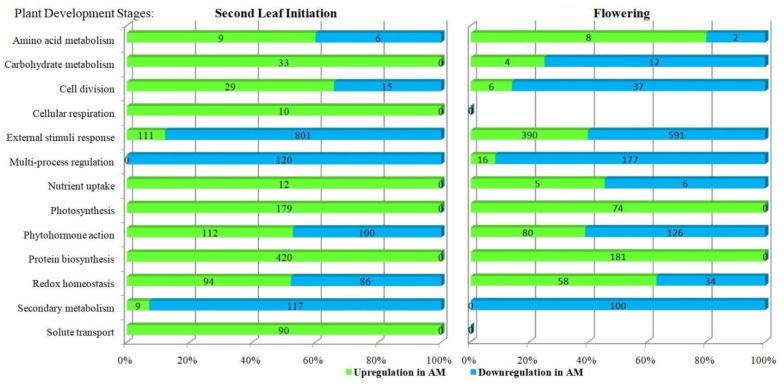
The principal classes of biological processes for leaf *M. lupulina* genes with up- and downregulation at second leaf and flowering stages under conditions of inoculation with *R. irregularis*.

In order to identify common patterns caused by the diversity of effects of *R. irregularis* on the *M. lupulina* leaf transcriptome, we analyzed the influence of AM fungus on the dynamics of the main classes of biological processes (functional categories were determined accordingly [41,53]). The effect was examined at the early vegetative and generative stages of the host plant. The most common functional categories (classes) are presented in Figure 4. It can be seen that in the stage of the second leaf, none of the classes from “Carbohydrate metabolism”, “Cellular respiration”, “Nutrient uptake”, “Photosynthesis”, “Protein biosynthesis”, and “Solute transport” contained groups of gene enrichment with downregulation. During the flowering stage, the number of fully regulated classes significantly decreased from 6 to 2 (these included only “Photosynthesis” and “Protein biosynthesis”). During the flowering stage, there are no upregulated genes in the “Cellular respiration” and “Solute transport” groups (typical for the early development), but genes with downregulation were also not found. The smallest group is the “Cellular respiration” group. The largest group is the group “External stimuli response”. The groups that most intensively changed the activity of expression, the accumulation of transcription products, are the groups “Multi-process regulation” (at the second leaf development stage; analyzed genes in this group were characterized by downregulation only) and “Secondary metabolism” (similarly, but only at the stage of flowering). During the transition from the early vegetative stage (second leaf development) to the beginning of the generative stage (flowering), there was a change from upregulation to downregulation of genes in the groups “Carbohydrate metabolism”, “Cell division”, “Nutrient uptake”, “Phytohormone action”. On the contrary, during the transition from the early vegetative stage to the beginning of the generative stage, an increase in the proportion of genes with downregulation was observed in the groups “External stimuli response”, “Redox homeostasis”.

## 3. Discussion

Recently, various *omics* approaches have been widely used to study the function of supra-organismal symbiotic PMS, which is formed during the development of arbuscular mycorrhiza [23,26,27,29,30,31,32,34,35]. These approaches include transcriptomic analysis, which identifies groups of DEGs that are significant for a plant upon interaction with AM fungus [33,34,35]. In the presented investigation, the MACE-Seq method was chosen [37]. It was applied to the efficient “*M. lupulina* + *R. irregularis*” PMS.

### 3.1. Diversity in the Effects of R. irregularis on M. lupulina Leaf Transcriptome Is Defined by High Response of MlS-1 Plants to AM and Adaptation to Phosphate Starvation

The *M. lupulina* MlS-1 line is obligate micotrophic under conditions of low phosphorus (P) level in the substrate and has a high level of MGR (Figure S1A) and mycorrhiza activity (Figure S1B,C). The AM frequency (*F*) increased to almost 90% at the flowering stage of the host plant, and the abundance of arbuscules (a) decreased to less than 40%, which is consistent with our earlier investigations of this line and is, probably, associated with an increased role of intraradical mycelium in metabolic processes at the late stages of AM development [47,48]. A similar study, using MACE-Seq to analyze AM plants’ symbiotic genes, was implemented on *Pisum sativum* [41]. The symbiotic efficiency (EIBSM) using plant lines inoculated with *R. irregularis* was much lower (~25–50%) [45]. The high AM efficiency (MGR) and the high AM activity of the MlS-1 line allowed us to identify a bigger number of DEGs by the factor of the influence of plant mycorrhization both at the early and late stages of AM symbiosis development (Figure 2). The most significant differences were detected by the mycorrhization factor (Figure 1) in comparison with less clear differences obtained by the development stage factor. This could be expected under conditions of low P level in the substrate, since the analyzed MlS-1 line has an ultra-high response to mycorrhization. The low P level determines the highest symbiotic efficiency of AM (MGR), which can exceed 300% calculated both by the dry biomass and by P content in the aboveground parts of the *M. lupulina* MlS-1 line [46] used in our study. At a higher P level, the effect of AM inoculation on gene expression would probably be much lower, since the increased P level reduces MGR (symbiotic efficiency) and root mycorrhization parameters in most cultivated plants: *M. truncatula* [54], *Capsicum annuum*, *Zea mays*, *Cucurbita pepo* [55], and other species [56]. On the other hand, the specific mycorrhization markers can demonstrate a stronger influence in PCA.

In *M. lupulina* leaves, according to our data, 1689 and 1608 genes were upregulated, but 1545 and 1710 genes were downregulated at the second leaf and at the flowering stages, respectively. Analysis of the literature data showed that, unlike in roots, the knowledge about DEGs in *Medicago* leaves, which depend on AM inoculation and P level, is very poor. Nevertheless, both factors intensively affect the biological processes in the aboveground plant organs. For example, the results of L. Adolfsson et al.’s [57] study revealed the effect of *R. irregularis* inoculation on the transcriptome and metabolome of *M. truncatula* leaves. The AM efficiency was only 20% (insignificant efficiency) when calculated by total P in the shoots. This is probably the reason that only 297 AM-regulated genes were identified, i.e., 16 times less than in our study in the highly responsive *M. lupulina* MlS-1 line. Using single-strand RNA sequencing, it was shown that the total number of significant DEGs was an order of magnitude lower in *M. truncatula* shoots (<100 DEGs, *p* < 0.05) than in roots [58]. According to our data, the expression of >4500 genes (*p_adj_* < 0.01) was changed in *M. lupulina* leaves. In addition to higher AM efficiency, the advantage of the used PMS is more accurate analysis of alterations at the crucial stages of the initiation of the development of certain organs (second leaf or flower). Other model systems, such as “*Solanum lycopersicum* + *Funelliformis mosseae*” [59], “*Zea mays* + *Acaulospora tuberculata*” [60], “*Cucumis melo* + *R. irregularis*” [61], “*Nicotiana tabacum* + *Glomus etunicatum*” [62], etc., are mostly analyzed at relatively early stages of AM development. But these studies do not take into account the development stages of the host plant in the result discussion due to the use of “the day after inoculation/sowing” (DAI/DAS) as a standard in time-course experiments. Meanwhile, the development stages at the analyzed DAI/DAS of such plants differ significantly. It should also be taken into account that many biochemical changes under mycorrhization are extremely species-specific, even for closely related plant species [4]. On the other hand, in the single known study of the effect of *R. irregularis* on the *P. sativum* transcriptome by MACE-Seq, the presence of only 456 DEGs (*p_adj_* < 0.05) was shown [41], among which 180 genes were upregulated, and 276 were downregulated in *P. sativum* lines with high AM efficiency compared to lines with low efficiency. Our results with *M. lupulina* leaves showed that the variation in the level of gene expression was as follows. (1) In the group with base mean equal to 5000…50,000 fragments per million (FPM), the log_2_FoldChange was only from −1.20 to +1.35; (2) in the group with base mean 500…5000 FPM, it was −4.42…+2.60; (3) in the group with base mean 50…500 FPM, it was −5.45…+3.85; (4) in the group with base mean 5…50 FPM, it was −7.18…+5.79. Thus, the greatest difference in gene expression in the *M. lupulina* leaves was observed for genes with lower expression. It is noteworthy that in [41], the analyzed *P. sativum* genes belonged only to the group with 5…50 FPM. It might be assumed that our new data are of considerable novelty and important for revealing the mechanisms controlling the development of effective AM symbiosis. In consequence of the study, we had the opportunity to analyze a significant number of functional groups of “Gene Ontology” (GO). Enrichment was used for genes with AM upregulation and AM downregulation both at early and late stages of AM symbiosis development (Figure 3). In terms of GO, the selection of DEGs with the highest response to AM was carried out with a reliable expression level >5 FPM. As a result, we identified 244 of the most responsive potential marker genes for the development of efficient AM symbiosis, “*M. lupulina* + *R. irregularis*” PMS, belonging to different GOs and not duplicating each other (Tables S1 and S2). The greatest intensification in gene expression in plants with AM compared with plants without AM (upregulation) was 4.2 and 6.8 times at the second leaf and flowering development stages, respectively. (1) The *MtGA20ox1* ortholog (encodes gibberellin 20 oxidase and participates in gibberellin biosynthesis, according to [63]; GO:0016114) had strong upregulation both at the early and late stages of AM development; (2) the *Medtr4g027840* ortholog (encodes non-specific Lipid Transfer Protein, LTP, participates in the transfer of phospholipids/galactolipids across membranes, can inhibit the growth of fungi/bacteria, and acts as a positive regulator of plant resistance to diseases, according to [64]; GO:0009414) had significant upregulation at the flowering stage of *M. lupulina*, which is consistent with the data of [65] (upregulation in shoots in *M. truncatula sunn*, “super numeric nodules”, mutant at 21 DAS; *p*-value < 0.05). Downregulation of genes in AM plants was more pronounced than upregulation, with a maximum of 15.1 and 33.5 times at the second leaf and flowering development stages, respectively. (1) The *MtMYB100* ortholog (encodes MYB transcription factor 100; MYB family genes presumably participate in the reaction of *Sesbania cannabina* AM plants to salt stress [66] and participate in the development of legume–rhizobial symbiosis in *M. truncatula* and reactions to abiotic stress—cold, freezing, drought, salt [67]; GO:0042742) changed downregulation in *M. lupulina* to the high upregulation at the flowering stage, which was not previously shown in dynamics in other PMS; (2) the *MtSPX3* ortholog (encodes SPX domain-containing protein, regulates responses to P starvation, and controls the timely degradation of arbuscules [68]; GO:0016036) had downregulation at the early and especially at the late stage of AM symbiosis due to the inability of *M. lupulina* plants to adapt to P starvation without AM).

### 3.2. Functional Categories of Up- and Downregulated Genes by AM in M. lupulina Leaves

All genes of the “Cell division” functional category (Figure 4) had upregulation at the second leaf development stage, which determined the AM-dependent acceleration of the *M. lupulina* plants’ development. Particularly, the second leaf stage is characterized by intensive vegetative growth of the plant. It was found to be especially important for AM-induced adaptation to P starvation. With time, a plant switches from vegetative to generative development, which is accompanied by a certain decrease in the number of upregulated genes, possibly due to alterations in meristem activity and metabolic fluxes. In our study, at the flowering stage, genes with upregulation in the “Cell division” group were still present, but the number of genes with downregulation increased by 2.5 times (Figure 4). The literature data are limited, and there is no information about DEGs that coincide in GO with those identified in our work and expressed in leaves. But earlier it was shown that genes belonging to the GRAS family and playing a role in cell division (such as SHR, SCR, SCL3, etc.) were induced in roots of *Solanum lycopersicum* during mycorrhization with *R. irregularis* [69].

The changes in the “Cell division” group are in good agreement with the AM effect on the expression of genes of the “Protein biosynthesis” group (Figure 4). All the genes of this group were characterized by significant upregulation, and the group is the most numerous in terms of the number of upregulated genes at the second leaf stage. The analysis of protein biosynthesis genes showed an intensive upregulation of genes involved in the biosynthesis of small and large ribosomal subunits (SSU and LSU, respectively) at the second leaf stage. Previously, it was also shown that at this stage (24 DAS), the AM plants *M. lupulina* already have more developed leaves, accelerated development in comparison with control plants not inoculated with AM fungus, and enlargement in leaf surface area [29]. This coincided with the enhancement of photosynthetic processes in mycorrhized plants. At the flowering stage, the group still did not show downregulation of genes, but the number of upregulated genes shifted down significantly (2.3 times). The high expression of the genes gathered in the “Protein biosynthesis” group also confirms our assumption that mycorrhization provides better growth conditions with a better supply of mineral nutrients to the plant and thereby stimulates biosynthetic processes [29]. Meanwhile, in the less-responsive-to-inoculation system “*M. truncatula* + *R. irregularis*”, at 30 DAS, in the study of L. Adolfsson et al., it was shown that only a quarter of the genes were characterized by upregulation in the “Protein biosynthesis” group [57]. Nevertheless, during AM symbiosis development, usually, an increase in protein biosynthesis is observed; an increase in the content of proteins associated with photosynthesis was shown in the “*M. truncatula* + *R. irregularis*” system [70], and a higher protein content in leaves was detected in the “*Phaseolus mungo* + *Funneliformis mosseae*” and “*Phaseolus mungo* + *Acaulospora laevis*” systems [71]. These results are compatible with the intensification of cell division processes, since it is necessary to strengthen protein synthesis in order to form new organs and tissues.

Changes in the “Amino acid metabolism” group under mycorrhization included a number of genes with both up- and downregulation (Figure 4). The clustering of various groups of metabolites, discovered earlier, demonstrated the central role of amino acid–carboxylate and carbohydrate clusters at the early and late stages of *M. lupulina* development. A higher content of serine, methionine, histidine, and other nitrogen-containing compounds was detected in comparison with non-mycorrhized plants, as well as GABA, cyanoalanine, oxoproline, and arginine, at the second leaf development stage [29]. It is noteworthy that none of the amino acids had lower contents under AM at the same time at the early and late stages. The influence of AM is more probably species-specific. For example, in the “*Lotus japonicus* + *F. mosseae*” system (at the late development stages), the effect of AM on the level of organic acids, involved in the main pathways of catabolism and in the metabolism of amino acids, was negative [9]. However, for many plant species (*Plantago lanceolata*, *Plantago major*, *Veronica chamaedrys*, *Poa annua* [4], and *M. truncatula* [4,28]), an increase in the level of a number of amino acids and some fatty acids was reported. Coming back to transcription level, we will examine L. Adolfsson et al.’s findings. Mycorrhization triggered upregulation of the genes of the “Amino acid metabolism” group, such as *Medtr5g013470* (encoding glutamate dehydrogenase), *Medtr5g005670* (encoding tryptophan synthase beta chain), *Medtr7g086300* (encoding methionine synthase), etc. [57]. The elevation of the gene expression in the “Cellular respiration” group at the *M. lupulina* second leaf development stage is partly associated with changes in the “Amino acid metabolism” group. The *Medtr4g116350* ortholog (encoding putative protein chlororespiratory reduction 6) and the *Medtr6g015275* ortholog (encoding putative protein chlororespiratory reduction 7) were among this minor group. The strengthening of cellular respiration might indicate the transfer of energy to the intensification of various synthetic processes that occur outside the chloroplast. There is little information in the literature about the role of this group of genes in the development of AM symbiosis. However, the “*M. truncatula* (wt—cv. Jemalong A17) + *R. irregularis*” system shows upregulation of the *MT4Noble_001987* gene (encoding proton-translocating NADH-quinone oxidoreductase) [65]. But not all metabolic processes in *M. lupulina* leaf cells were characterized by AM-symbiosis upregulation. A detailed analysis of genes not grouped into single GO groups showed a multidirectional effect of AM on the elements of “Cellular respiration”. The genes involved in glycolysis and the tricarboxylic acid cycle (TCA cycle) were mainly downregulated under mycorrhization. It is consistent with results assessing the effect of AM on the metabolome of *M. truncatula* leaves [4] and other dicotyledonous plants [9,31,72]. The results are also confirmed by our data obtained earlier; AM significantly suppressed the activity of the TCA cycle and pathways associated with the exchange of carboxylates and amino acids [29].

The “Photosynthesis” group was expected to have a clear upregulation of DEGs, especially pronounced at the second leaf stage (Figure 4). AM fungi strongly depend on the photosynthesis products of the host plant; therefore, they have a great impact on this process [56]. We have previously shown that *R. irregularis* significantly increases both the content of chlorophyll a and b, and leads to a considerable increase in leaf area during the second leaf development stage, 24 DAS [49]. The importance of carbohydrate metabolism is confirmed by the results, which showed high photosynthesis activity in the presence of AM according to the assessment of the effective photochemical quantum yield of photosystem II—Y(II) at an early stage of development, with a sequential decrease at the flowering stage [49]. The results of the presented study indicate the strong effect of the AM fungus on photosynthesis. A substantial part of the genes (189 DEGs) involved in photosynthesis had intensive upregulation during mycorrhization: 61 genes with upregulation only at the second leaf stage, with a gene ratio (GR) equal to 0.47, 74 genes only at the flowering stage, with GR = 0.56, and 54 genes with upregulation at both stages, with GR = 0.32. These DEGs belonged to seven groups: GO:0009767, GO:0009768, GO:0009773, GO:0015979, GO:0015995, GO:0019684, GO:0022900. No GO groups involved in photosynthesis with AM-downregulation were found. Meanwhile, the genes involved in the photosynthesis process had one of the highest levels of expression in the leaves of *M. lupulina*, compared with the genes of other groups. For example, the *Medtr3g010000* ortholog encoding Photosystem I reaction center subunit V had an expression of >2000 FPM in non-mycorrhized plants and >4400 FPM in AM plants during the flowering stage. The following genes with expression >1000 FPM were also identified: the *Medtr5g098785* ortholog encoding light-harvesting complex I chlorophyll A/B-binding protein 3, and the *Medtr7g118290* ortholog encoding thylakoid membrane phosphoprotein 14 kDa protein. During the flowering stage, the *Medtr3g086230* ortholog (encoding photosystem II core complex protein psbY) was upregulated, involved in the regulation of photosynthesis with high expression (>3500 FPM). Enhanced expression of the “Photosynthesis” group genes has been shown in a number of PMS, but this is not always typical. For example, in the study of K.R. Cope et al., the effects of the efficient AM fungus *R. irregularis* and the inefficient AM fungus *Glomus aggregatum* on the transcriptome of *M. truncatula* were analyzed. Data indicated that only the efficient *R. irregularis* fungus stimulated the expression of genes associated with “Photosynthesis” in shoots: *Medtr1g015290* (encoding photosystem II reaction center X protein, PsbX); *Medtr2g082580* (encoding oxygen-evolving enchancer protein); *Medtr1g115410* (encoding photosystem II reaction center PsbP family protein); *Medtr1588s0010* (encoding ATP synthase F1, gamma subunit); *Medtr6g084320* (encoding ABC transporter C family protein) [58]. Also, the “*M. truncatula* (wt) + *R. irregularis*” system revealed upregulation of the following photosynthesis genes: *Medtr2g020310, Medtr4g050860, Medtr3g035600, Medtr4g019000, Medtr4g050880, Medtr4g050850, Medtr1g112050* [65]. In *M. truncatula* studies, no more than 16 genes with upregulation and 30 genes with downregulation under *R. irregularis* inoculation were found, which is two orders of magnitude less than in with the efficient “*M. lupulina* + *R. irregularis*” PMS model.

Dramatic changes were revealed in the “Carbohydrate metabolism” group, with a shift of upregulation to the dominance of downregulated genes in AM plants (Figure 4). Probably, this process is tightly regulated at the late stage of host plant development. Genes with up- and downregulation were also found in other PMS. In the study of L. Adolfsson et al., the “Carbohydrate metabolism” group was not determined, but it was mentioned that ⅔ (68%) of the genes of the “Primary metabolism” group had upregulation, including some genes of the “Carbohydrate metabolism” group [57], for example, *Medtr6g012380* and *Mtr.6550* (encoding granule-bound starch synthases). The system “*M. truncatula* (wt) + *R. irregularis*” also demonstrated upregulation of genes involved in “Starch and sucrose metabolism”: *Medtr2g020240* and *Medtr4g099110* [65]. These data are in agreement with the results of a previously provided metabolic analysis of *M. lupulina* in the same PMS model. A delay in sugar accumulation was observed in comparison with plants without AM. Such a decrease in the accumulation of carbohydrates in leaves may be an indicator of intensive outflow of these metabolites into mycorrhized roots. The conclusion is that it could be the cause of starch synthesis slowing down in comparison with non-mycorrhized plants [49].

The “Secondary metabolism” group had substantial downregulation by AM-symbiosis, especially for phenols in *M. lupulina* leaves. Previously, the analysis of the metabolome showed that the reduced content in the “ + AM” *M. lupulina* leaves had suberyglycine at the second leaf development stage and phenol_RI = 2913 at the flowering stage [49]. Similarly, the “*M. truncatula* + *R. irregularis*” PMS, the “Secondary metabolism” group, such genes as *Medtr8g040780* (encoding anthranilate N-benzoyltransferase) and *Medtr2g025120* (encoding 1-aminocyclopropane-1-carboxylate oxidase) were downregulated [57]. Information about the effect of AM on the level of terpenoids in the literature is contradictory. The expression of genes involved in the terpenoid biosynthesis pathway was often enhanced with AM, but there was a general decrease in terpenoid content, and the content of glycosylated zhanic acid derivative was higher with AM [57]. In addition, AM elevated the level of terpenoids in plants of different PMS, such as “*Ocimum tenuiflorum* + *R. intraradices*”, “*Thymus vulgaris* + *F. mosseae*”, “*Coriandrum sativum* + *Glomus hoi*”, “*Satureja macrostema* + *R. irregularis*”, “*Salvia officinalis* + *R. clarus*”, “*Lavandula angustifolia* + *R. intraradices*”, as well as the level of phenols in plants of PMS, including “*Passiflora alata* + *Acaulospora longula*”, “*P. alata* + *Gigaspora albida*”, “*Eclipta prostrata* + *Septoglomus deserticola* + *F. mosseae* + *Acaulospora lacunosa*” [73]. Such modulation of terpenoid balance might mirror its important role in the regulation of plant growth and protection from stress factors [57,74].

The activity of plant metabolism depends on mineral nutrition and water supply. The efficient plant–microbe interaction is aimed at accelerating those processes. But the mechanisms may vary widely. Thus, in the roots, there is a direct increase in P nutrition due to, for example, the functioning phosphate transporters specific to AM [56]. However, in leaves under P starvation conditions, reverse regulation can be observed for a number of genes, since specific P transporters for AM have not been found in these organs. In the “*M. lupulina* + *R. irregularis*” PMS, the “Nutrient uptake” group contained upregulated genes at the second leaf stage, but at the flowering stage, the ratio of genes with up- and downregulation was equal. It was revealed that the *Medtr7g095430* ortholog (encoding sulfate transporter) was upregulated at both development stages, the *Medtr5g032420* ortholog (encoding dicarboxylate carrier protein and participating in sulfate transmembrane transport) was upregulated at the flowering stage, and the *Medtr7g083790* ortholog (encoding phosphate carrier protein) was downregulated at the flowering stage. A positive AM effect on sulfate absorption, as well as a decrease in the sensitivity of AM plants in the “*M. truncatula* + *R. irregularis*” PMS to sulfur deficiency, had already been shown [75]. On the other hand, AM-dependent transport of nutrients from the roots resulted in an intensive accumulation of phosphates in the form of glycerophosphoglycerol and inorganic phosphate in the leaves of AM plants due to active P uptake involving the symbiotic transporter MlPT4 in the roots [49,50]. The “Solute transport” group associated with nutrient uptake consisted of genes characterized by significant upregulation at the second leaf stage. The main GO groups were the following: (1) water transport, GO:0006833 (aquaporin genes of the MIP family: orthologs *Medtr1g095070*, *Medtr3g118010*, *Medtr6g033010*, *Medtr8g013680*, *Medtr1g006490, Medtr7g101190*, *Medtr8g098375*, *Medtr5g010150*, *Medtr3g070210*, *Medtr4g059390, Medtr4g006730*, *Medtr2g104940*, etc.). Similarly, in the study of G. Quiroga et al., in the “*Zea mays* + *R. irregularis*” PMS, the relationship between upregulation of aquaporin gene expression (*ZmPIP1;6*, *ZmPIP2;2* and *ZmTIP4;1*) and drought resistance of the host plant was estimated [76]: (2) urea transport GO:0015840 (the *Medtr1g079760* ortholog encoding ammonium/urea transporter, etc.). Interestingly, the expression of the *Medtr1g079760* ortholog at the flowering stage was even higher in the leaves of plants with AM compared to plants without AM. These data are consistent with the results of the analysis of the leaf metabolome, indicating a higher urea content at the flowering stage [49]. The role of aquaporins in the development of a plant organism is difficult to overestimate, as well as the positive effect of AM fungus on the expression of genes encoding aquaporins, and therefore, this subject deserves separate investigation in subsequent studies.

The “Reaction to external stimuli” group was the largest one. It included genes that play a role in the adaptation of plants to environmental changes. The number of upregulated genes in this group increased (3.5 times) from the early stage to the late development stage. In the “Reaction to external stimuli” group, the gene with the greatest downregulation during mycorrhization in our experiment was identified. The expression of the *MtSPX3* ortholog (encoding a protein of the SPX family containing the SPX domain) in the leaves of AM plants was 33.5 times lower than in the control during mycorrhization at the flowering stage. Interestingly, this particular gene with the highest response among 244 selected genes belongs to the group GO:0016036, “cellular response to phosphate starvation” (Table S2). Further analysis of this entire group revealed a significant number of genes with a high response to P starvation in plants without AM. But the plants with AM characterized by a lower level of gene expression in GO:0016036 were probably adapted to these P conditions, which caused a high downregulation of the genes of the GO:0016036 group (Table S3). Moreover, the reaction to P starvation increased with the development of the plant by an average of 2.0 times for 24 analyzed genes from the early stage (second leaf) to the late stage of symbiosis development (flowering). According to C. Hsieh et al.’s data (“*Oryza sativa* + *R. irregularis*” PMS), 24 genes of the GO:0016036 group were downregulated in shoots [77]. However, the set of genes is different, and the response to AM is much weaker (2–4 times) than in our “*M. lupulina* + *R. irregularis*” PMS. According to M. Karlo et al. [65], in the “*M. truncatula* (wt) + *R. irregularis*” PMS at 21 DAS, there was also suppression of gene expression in the GO:0016036 group: the *MtDMPP950* ortholog (encoding 2,3-diketo-5-methylthio-1-phosphopentane phosphatase) and the *MtPHO1.3* ortholog (encoding the phosphate transporter 1.3). However, a *M. truncatula sunn* mutant also showed downregulation of *MtDMPP950, MtPPC1* (encoding phosphoenolpyruvate carboxylase), *Medtr1g067380* (encoding phosphatidate phosphatase), *Medtr1g080020* (encoding phosphoenolpyruvate carboxylase-related kinase), and *MtUGP* (encoding UTP-glucose-1-phosphaturidyltransferase) [65]. In another experiment in “*M. truncatula* + *R. irregularis*” PMS under conditions of two Pi levels, it was also discovered that AM inoculation caused changes in the transcriptome of the leaves. But results were different and often unrelated to the influence of the P supply factor. Furthermore, microarray-based gene expression analysis revealed a slight intersection of DEGs in plant leaves of AM variant and P variant (32 out of 698 genes, i.e., approximately 5%) [57]. Furthermore, the genes identified in our study (*MtPLD15*, *Medtr4g115920*, *Medtr4g015260* orthologs), assigned to the GO:0016036 group, were also downregulated under P fertilizer supply in the study of L. Adolfsson et al. [57]. According to other experiments, the *Medtr1g083620* gene also belonged to the group of DEGs in the leaves of AM-inoculated *M. truncatula*, regardless of the presence or absence of sulfur deficiency stress [75]. Thus, it is not yet possible to classify these genes as AM- or P-regulated. On the other hand, the comparison with the list of genes responsive to P deficiency in the *M. truncatula* leaves, presented by T. Wang et al. [78], did not reveal a single match with DEGs for the genes in GO that we selected as the main ones in the GO groups (244 groups; Table S2) and among the 24 genes of the GO:0016036 group “cellular response to phosphate starvation” (Table S3). Therefore, the species-specific reactions in response to AM and P starvation are supposed to be very different. Nevertheless, among the genes of the GO:0016036 group with downregulation identified in the “*M. lupulina* + *R. irregularis*” PMS, the WRKY transcription factor gene (the *MtWRKY6* ortholog) and the MYB-HB-like transcription factor genes (the *MtNIGT1* and *Medtr7g098250* orthologs) were found. Their role in the response to P starvation has already been shown in *M. truncatula* and *Phaseolus vulgaris* [79,80]. In the “*Sesbania cannabina* + *F. mosseae*” PMS, the role of WRKY and MYB transcription factors in the response of AM plants to salt stress, which was accompanied by increased photosynthesis and absorption of reactive oxygen species (ROS), was shown [66]. Microarray-based transcription analysis was performed in *M. truncatula* leaves with *R. intaradices* inoculation and revealed systemic gene induction, including upregulation of the WRKY transcription factor in response to P starvation [81]. Further comparison of the DEGs obtained in our experiments in the “*M. lupulina* + *R. irregularis*” PMS (Tables S2 and S3) and the DEGs obtained by L. Adolfsson et al. in the “*M. truncatula* + *R. irregularis*” PMS showed a number of similarities. For example, upregulation of the *CSD1* ortholog (encoding superoxide dismutase [Cu-Zn] protein) at the flowering stage, upregulation of the *Medtr6g059680* ortholog (encoding kunitz-type trypsin inhibitor-like 2 protein) at both tested time points, downregulation of the *FER1* ortholog (encoding ferritin 1) at both time points, as well as the *FER3* ortholog (encoding ferritin 3) were fixed. Ferritin binds Fe(III) in a non-toxic form in plastids [82]. In L. Adolfsson et al. [57], the *Medtr5g083170* (encoding ferritin 2), *FER1,* and *FER3* also were downregulated. At the same time, the last two genes were also characterized by downregulation in *M. truncatula* [65]. In the efficient “*M. truncatula* + *R. irregularis*” PMS, the downregulation in the “Abiotic stress” group was observed as well: *FER1*; *Medtr1g048990* (encoding superoxide dismutase); *Medtr8g059170* (encoding NAC transcription factor-like protein) [58]. On the other hand, the upregulation in the “Biotic stress” group was not observed, whereas it was characteristic for the inefficient “*M. truncatula* + *Glomus aggregatum*” PMS for certain genes: *Medtr4g054920* (encoding cytochrome P450 family 94 protein); *Medtr4g120760* (encoding pathogenesis-related protein bet V I family protein); *Medtr5g053950* (encoding allene oxide cyclase); *Medtr8g096900* (encoding pathogenesis-related thaumatin family protein); *Medtr4g092010* (encoding (3S)-linalool/(E)-nerolidol/(E,E)-geranyl linalool synthase) [58].

The “Multi-process regulation” group included a significant number of genes, usually with downregulation in the main GO groups (Figure 4). Its analysis is quite problematic due to the multiplicity of processes they are involved in. There are few examples in the literature of combining genes into a common “Miscellaneous” group, which members mostly had preferential downregulation (63%) [57]. The explanation is based on their higher expression in non-mycorrhized *M. lupulina* plants as the response to P starvation and adaptation of AM plants to developmental conditions. However, additional investigation is required to analyze this group of genes.

Analysis of the genes of the “Redox homeostasis” group showed that more than half of them had upregulation in the “*M. lupulina* + *R. irregularis*” PMS. The increased level of ROS is known as response to stress factors [83]. The main source of ROS in mycorrhized root cells is NADPH oxidase encoded by *MtRbohE* in *M. truncatula* [84]. The main antioxidant molecules in this case can be glutathione (reduced form—GSH), ascorbate (AsA), proline, carotenoids, and α-tocopherols, which are necessary to maintain the redox status in plant cells [85]. A. Liu et al. [86] observed that in the “*Solanum lycopersicum* + *F. mosseae*” PMS, the content of AsA and GSH was higher than in the roots of non-mycorrhized plants. The expression levels of the corresponding genes involved in antioxidant protection such as *MPX* (encoding ascorbate peroxidase), *MDHAR* (encoding monodehydroascorbate reductase), *DHAR1* (encoding dehydroascorbate reductase), and *GR* (encoding glutathione reductase) were higher in roots of AM plants. It has been shown that inoculation of the *Cajanus cajan* plant by *R. intraradices* enhanced the mechanisms of antioxidant protection, including the ascorbate–glutathione cycle in the roots under conditions of increased nickel content [87]. DEGs detected in the roots of *Casuarina glauca* plants inoculated with *R. irregularis* under salinization conditions included a significant number of antioxidant enzyme genes [88]. The mechanisms of antioxidant protection in the leaves of AM plants have not been studied enough. For example, in the “*M. sativa* + *R. intraradices*” PMS, a higher content (almost 2 times both in shoots and roots) of such antioxidant enzymes as superoxide dismutase, catalase, and ascorbate peroxidase during mycorrhization was shown in comparison with the variant without inoculation [89]. In the “*Bambax ceiba* + *R. irregularis*” PMS, the activity of antioxidant enzymes in leaves (ascorbate peroxidase, glutathione peroxidase, dehydroascorbic acid in drought and well-watered treatment; superoxide dismutase, dehydroascorbate reductase in drought) was higher in comparison with non-mycorrhized plants [90]. In our efficient “*M. lupulina* + *R. irregularis*” PMS, the upregulation at both the second leaf development stage and flowering stage in leaves was shown for genes of the “Redox homeostasis” group, such as *Medtr3g089065*, *Medtr7g093490*, *Medtr1g114290* orthologs encoding one protein—thioredoxin (Table S2). It was previously reported that superoxide dismutases [91], glutaredoxins [92], etc., participate in antioxidant protection in AM symbiosis. Probably, thioredoxin, with the participation of thioredoxin reductase, can also play an active role as an antioxidant [93] in this PMS. Analysis of the transcriptome of roots (not leaves) in the “*Medicago sativa* + *F. mosseae*” PMS under conditions of treatment by herbicide (atrazine) revealed higher resistance to stress due to increased expression of genes encoding thioredoxin, glutaredoxin, and guaiacol peroxidase as antioxidants absorbing ROS [94]. Thus, it should be assumed that in our study in the “*M. lupulina* + *R. irregularis*” PMS, the upregulation of three thioredoxin genes in leaves was shown for the first time both at the early and late stages of AM symbiosis development.

The results showed a multidirectional effect of *R. irregularis* fungus AM inoculation on the hormonal status of *M. lupulina* plants. In the “Phytohormone action” group, the upregulation in GO groups, such as “auxin homeostasis”, “auxin efflux”, and “response to cytokinin”, both at the second leaf development stage (at 24 DAS) and flowering stage (at 48 DAS) should be noted, while downregulation was revealed for the genes of “systemic acquired resistance, salicylic acid mediated signaling pathway” and “jasmonic acid mediated signaling pathway” both at the second leaf development stage and flowering. The dynamics of expression of genes responsible for the balance and mechanisms of auxin and cytokinin action are consistent with the results obtained by us using the enzyme immunoassay method in studying the effect of AM inoculation of the *R. irregularis* fungus on the content of auxin and cytokinins (such as zeatin and zeatin riboside) in the *M. lupulina* leaves [47,48]. Higher levels of accumulation of transcripts of cytokinin metabolism genes (two genes encoding cytokinin-O-glucosyltransferase—*Medtr2g035020* and *Mtr.13700*) were discovered in the *M. truncatula* leaves inoculated with AM [57]. Interestingly, in contrast to the efficient PMS used in our case, the formation of inefficient AM symbiosis in *M. truncatula* (at 28 DAS) affected genes involved in the biosynthesis of jasmonic acid (JA). At the same time, increased expression of *MYC2*, the main regulator of JA-dependent reactions, was detected, but the content of JA and salicylic acid in the leaves of AM plants was significantly reduced in comparison with that in control plants without AM [57]. Thus, it can be concluded that the hormonal system of plants reacts very subtly to the development of efficient symbiosis and requires further study using a comprehensive analysis of both inefficient and highly efficient lines, such as the *M. lupulina* MlS-1 line.

## 4. Materials and Methods

### 4.1. Plant and Fungus Biomaterials

Selected PMS, including the *Medicago lupulina* MlS-1 line, characterized by a high mycorrhiza growth response (MGR, AM efficiency) under conditions of low Pi levels in the substrate [30,46], as well as the efficient *Rhizophagus irregularis* RCAM00320 strain (Laboratory #4 of Ecology of Symbiotic and Associative Rhizobacteria, All-Russia Research Institute for Agricultural Microbiology, ARRIAM; the strain was previously known as the CIAM8 *Glomus intraradices* strain Shenck&Smith), forming highly efficient AM symbioses with most agricultural crops [95,96,97] and identified by the authors [98], were used. *R. irregularis* is an obligate symbiont; therefore, the culture of AM fungus was grown in *Plectranthus australis* (Laboratory #4, ARRIAM, Pushkin, St. Petersburg, Russia). The preparation of inoculant was described earlier [49]. For inoculation of one *M. lupulina* seedling, fragments of the *Plectranthus australis* roots containing ~100 vesicles of the AM fungus were used.

### 4.2. Experimental Design and Plant Growth Conditions

As a substrate, a mixture of soil:sand (2:1) was used for the cultivation of PMS in the pot experiment. Agrochemical soil characteristics: sod-podzolic loamy soil with a very low content of Pi (P_2_O_5_—23 mg/kg); K_2_O—78 mg/kg; organic matter content—3.64%; pH_KCl_—6.4, pH_H2O_—7.3. The substrate was autoclaved at +134 °C, 2 atm. for 1 h with repeated autoclaving after 2 days. Detailed procedures for experimental design were described earlier [30,49]. *M. lupulina* seeds were scarified for 5 min in concentrated H_2_SO_4_. Then, the seeds were stratified in Petri dishes for 1 day at +5 °C, and then germinated for 2 days at +27 °C in the dark. Seedlings of the same size were grown in a soil–sand substrate. The plants were inoculated once at this very early stage of development (seedling). Half of the plants were inoculated with AM-inoculant simultaneously with planting; the other half were not treated with AM-inoculant as a control. The plants were grown 2 seedlings in one pot filled with 210 g of soil–sand substrate. Watering of plants was carried out every other day up to 0.6 of the saturated water content. The growing protocol using UV-sterilized light phytobox was described earlier [47]. The micro-vegetative method provided optimal conditions for the development of AM and allowed us to avoid spontaneous infection with rhizobia and other symbiotic microorganisms. The plant leaves and roots were harvested twice: (1) at the 24th day after sowing and inoculation (DAS), at the 2nd real leaf development stage in plants with AM and plants without AM; (2) at the 48th DAS at the stage of flowering in plants with AM and plants without AM. Control plants were analyzed at the same time points. In general, at the 24th day, plants were characterized by acceleration of vegetative type of development, while at the 48th day, plants passed transiently from the vegetative to the flowering stage of development. Molecular genetic and microscopic analyses of plants were carried out for these 2 stages. The leaves of 8 plants were collected for 1 biological repeat (4 biological repeats per 1 treatment variant), weighed, frozen in liquid nitrogen, and then stored at −80 °C.

### 4.3. RNA Extraction, Library Preparation, and Sequencing

The plant material was snap-frozen in liquid nitrogen then ground using a mortar and pestle. RNA was isolated using the RNAzol reagent (MRC, Cincinnati, OH, USA) according to the manufacturer’s protocol. The RNA quality was assessed using the TapeStation system 4150 (Agilent, Santa Clara, CA, USA), software revision 3.1.1. The RNA concentration was measured using a Qubit Fluorometer and Qubit RNA BR Assay Kit (Thermo Fisher Scientific, Waltham, MA, USA). The MACE (Massive Analysis of cDNA Ends) libraries were generated using the Rapid MACE kit (GenXPro GmbH, Frankfurt, Germany) according to the supplier’s protocol. Library sequencing on an Illumina HiseqXTen platform (San Diego, CA, USA) was performed by Macrogen (Seoul, Republic of Korea). The MACE method used is limited to obtaining transcripts of nuclear genes and does not include the transcripts of the genes of the mitochondrial and chloroplast genomes.

### 4.4. Bioinformatic Analysis

The FastQC (version 0.11.8) [99] and multiqc [100] were used to assess the quality of raw reads. Adapters, low-quality reads, human and bacterial contaminants were removed as in [35], then the reads were deduplicated and cleaned of the TrueQuant adapter using seqkit (v. 2.2.0) [101]. The reads were mapped to the reference genome of *Medicago truncatula* (v5. [52]) and the gene expression was measured using STAR (version 2.7.3a) [102] with the -quantMode GeneCounts option enabled. The data analysis was performed in the R environment (v. 4.1.1). The sample clusterization was performed using pvclust package (v. 2.2) [103]. The differential gene expression analysis was performed using the DESeq2 [104] package in the R environment, and only the genes with adjusted *p*-value < 0.01 were considered significantly differentially expressed. The MapMan4 online tool [105] was used to assign functional categories to the differentially expressed genes. GO terms for genes of interest were obtained using the eggNOG-mapper program [106]. GO enrichment analysis (using the Kolmogorov–Smirnov test) and further visualization were performed in the topGO package [107], and the adjusted *p*-value of 0.05 was considered significant for these tests. The UpSetR [108] and ggplot2 [109] packages were used for visualization. The Mercator online tool, relying on curated reference classifications originating from a number of organisms (*Arabidopsis thaliana*, *Chlamydomonas reinhardtii*, *Oryza sativa,* and various other plant species [41,53]), was used to assign functional categories (classes) to DEGs. Principal Component Analysis (PCA) for leaf transcriptome profiles of mycorrhized and non-mycorrhized plants sampled at the 24th and 48th DAS was performed with pcaMethods using the prcomp command in R [110].

### 4.5. Data Availability

The sequence data have been uploaded to the NCBI database. The BioProject number is PRJNA873716, and biosamples used in this study are SAMN30499749-SAMN30499752.

### 4.6. Evaluation of Mycorrhization Parameters

The roots were dried at room temperature, then macerated and stained with trypan blue [111]. Mycorrhization indices were calculated [112]: *F*—the frequency of mycorrhizal infection in the roots; a—the abundance of arbuscules in mycorrhized parts of roots. Microscopic analysis of AM development was carried out using the computer program for calculating mycorrhization indices of plant roots, developed by A.P. Yurkov et al. [113].

### 4.7. Evaluation of Mycorrhizal Growth Response—AM Symbiotic Efficiency

The mycorrhizal growth response (MGR, AM efficiency) was calculated as the fresh weight of the aerial parts (or roots), using Odum’s formula:

E = ([+AM] − [−AM]) × 100%/[−AM],

where E is the AM symbiotic efficiency (MGR); [+AM] is the value of mycorrhized plant weight; [−AM] is the value of the weight of plants without AM. One-way ANOVA and Tukey’s HSD test (*p* < 0.05) as a post hoc test were used to compare differences in AM efficiency and mycorrhization parameters at different stages of plant development.

## 5. Conclusions

The study of the “*Medicago lupulina + Rhizophagus irregularis*” model system, characterized by high MGR, using the NGS method (MACE-Seq), revealed the significant variety of the effects of AM fungus on the *M. lupulina* leaf transcriptome. The analysis of 244 functional groups of “Gene Ontology” from 13 main functional classes, including genes presumably involved in the development of effective AM symbiosis, was carried out. At the early stage (the stage of second leaf development), the functional class “Cell division” included genes characterized by upregulation. However, the number of genes of this class with downregulation increased by 2.5 times during the subsequent transition of the plant from vegetative to generative development (at the flowering stage). These results are in good agreement with changes in the expression of genes of the other two classes “Protein biosynthesis” and “Photosynthesis”, which are also characterized by substantial upregulation at the early stage. This effect can be explained by the intensification of protein synthesis and carbohydrate accumulation required for the intensification of the formation and development of new organs and tissues as a result of AM symbiosis. At the later stage, the number of genes in these groups decreases by more than 2 times. At the same time, no GO group involved in photosynthesis and characterized by downregulation in mycorrhized plants was detected. On the contrary, in the “Carbohydrate metabolism” class, there was a shift of gene upregulation to downregulation that may be due to the transport of carbohydrates from source (leaves) to sink (mycorrhized roots) organs. A completely different dynamic was revealed for the genes of the most numerous among the studied groups, “External stimuli response” (included 24 genes). Genes of the GO:0016036 group (“cellular response to phosphate starvation”) were characterized only by downregulation, both at the early and late stages of host plant development. It can be assumed that this effect is based on increased resistance to stress factors, including phosphorus starvation. Our study revealed that GO groups in the “Phytohormone action” class, such as “systemic acquired resistance, salicylic acid mediated signaling pathway” and “jasmonic acid mediated signaling pathway”, were also characterized only by downregulation in both stages of plant development. This may be due to the fact that these phytohormones are involved in elevation of phytoimmunity and resistance to a number of abiotic stressors. In conclusion, we will focus on a group of genes associated with redox status. In our study, for the first time, the upregulation of three genes encoding thioredoxin (“Redox homeostasis” class) in leaves of both tested stages of AM symbiosis development was shown. It can be assumed that *R. intraradices* treatment enhances the mechanisms of antioxidant protection in *M. lupulina* reducing the level of ROS that is increased under conditions of the stress factor of low Pi level.

Thus, the revealed dynamic changes in the expression of a large group of genes (affecting a whole range of processes) indicate greater stability of the “plant + AM microorganism” system. These genes can be considered as markers, since some were identified in a model effective symbiotic pair under conditions of the most intensive development of this efficiency, phosphorus starvation.

## Data Availability

Not applicable.

## Supplementary Materials

The following supporting information can be downloaded at: https://www.mdpi.com/article/10.3390/plants12203580/s1, Figure S1. Fresh weight of aboveground parts and roots of *M. lupulina* MlS-1 line with and without *R. irregularis* inoculation (A), F—the frequency of mycorrhizal infection in *M. lupulina* roots with *R. irregularis* (B); a—the abundance of arbuscules in mycorrhized parts of *M. lupulina* roots with *R. irregularis* (C). “APFW” and “RFW” is fresh weight of aboveground parts and roots, accordingly. “−AM” and “+AM” is the variant without and with AM fungus inoculation, accordingly. “+58%*” is significant (*p* < 0.05) mycorrhizal growth response (MGR, AM efficiency), and different letters (“a”, “b”) indicate significant differences within the same mycorrhizal parameter (ANOVA and Tukey’s test; *p* < 0.05). Figure S2. GO: Molecular function for leaf *M. lupulina* genes with different regulations. (A) Upregulation at both 2nd leaf formation and flowering stage under conditions of inoculation with *R. irregularis* (see ① in Figure 2). (B) Downregulation at both 2nd leaf formation and flowering stage under conditions of inoculation with *R. irregularis* (see ② in Figure 2). (C) Upregulation at 2nd leaf formation and downregulation at flowering stage under conditions of inoculation with *R. irregulari*s (see ③ in Figure 2). Figure S2. GO: Molecular function for leaf *M. lupulina* genes with different regulations. (D) Upregulation at 2nd leaf formation stage under conditions of inoculation with *R. irregularis* (see ⑤ in Figure 2). (E) upregulation at flowering stage under conditions of inoculation with *R. irregularis* (see ⑥ in Figure 2). (F) Downregulation at 2nd leaf formation stage under conditions of inoculation with *R. irregularis* (see ⑦ in Figure 2). (G) Downregulation at flowering stage under conditions of inoculation with *R. irregularis* (see ⑧ in Figure 2). Table S1. Main GO groups and functional classes of genes expressed in leaves of efficient *M. lupulina* MlS-1 line inoculated by *R. irregularis.* Table S2. Putative marker genes for the development of efficient AM symbiosis in “*M. lupulina* + *R. irregularis*” plant microbial system. Table S3. Downregulated genes of GO:0016036 group (cellular response to phosphate starvation) in *M. lupulina* leaves under condition of inoculation with *R. irregularis*.
