# Supplementary figures and images for: The Effects of Rhizophagus irregularis Inoculation on Transcriptome of Medicago lupulina Leaves at Early Vegetative and Flowering Stages of Plant Development
Source: Plants (Basel). 2023 Oct 15;12(20):3580. doi: 10.3390/plants12203580 (PMC10610208; doi:10.3390/plants12203580)

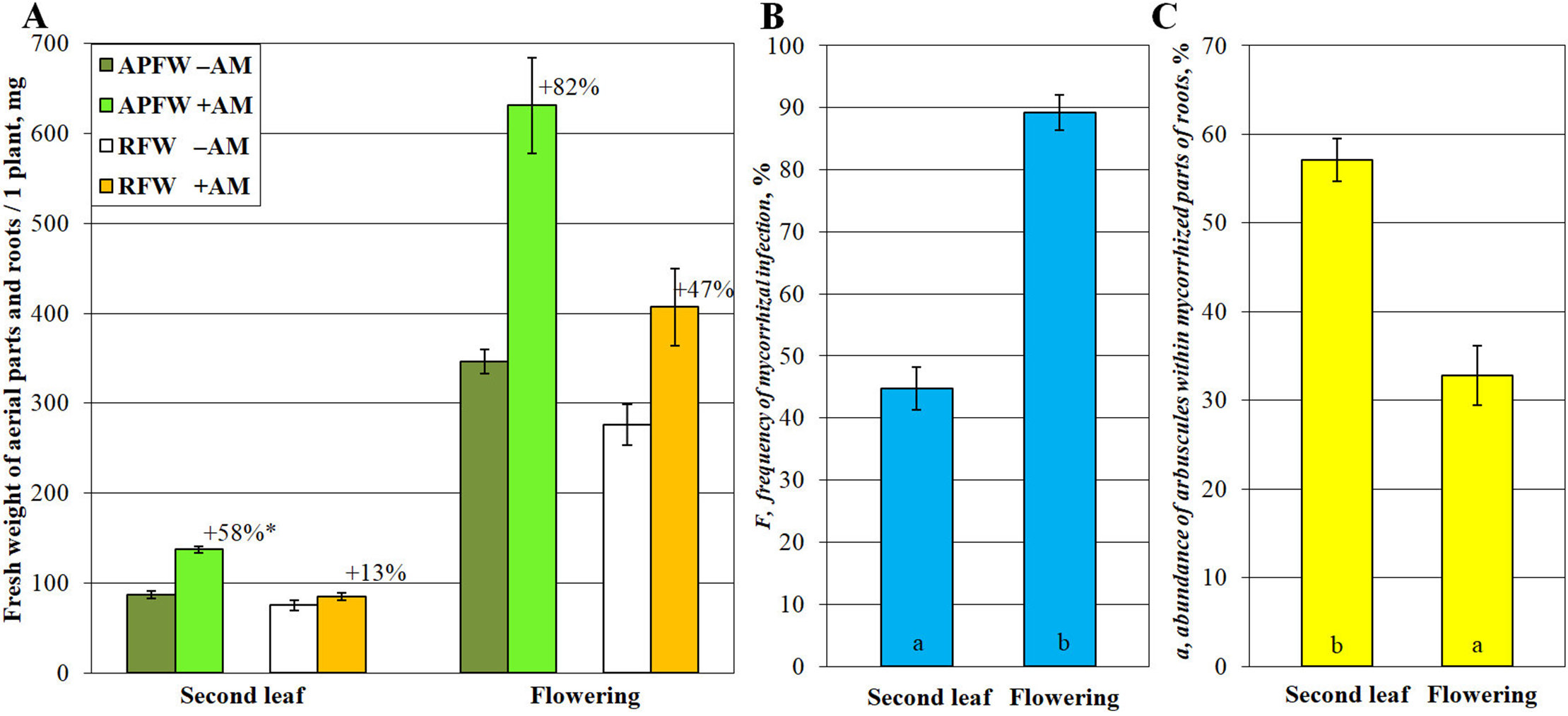

Supplement: Supplementary file 1 [file plants-12-03580-s001.zip › Figure S1.jpg]

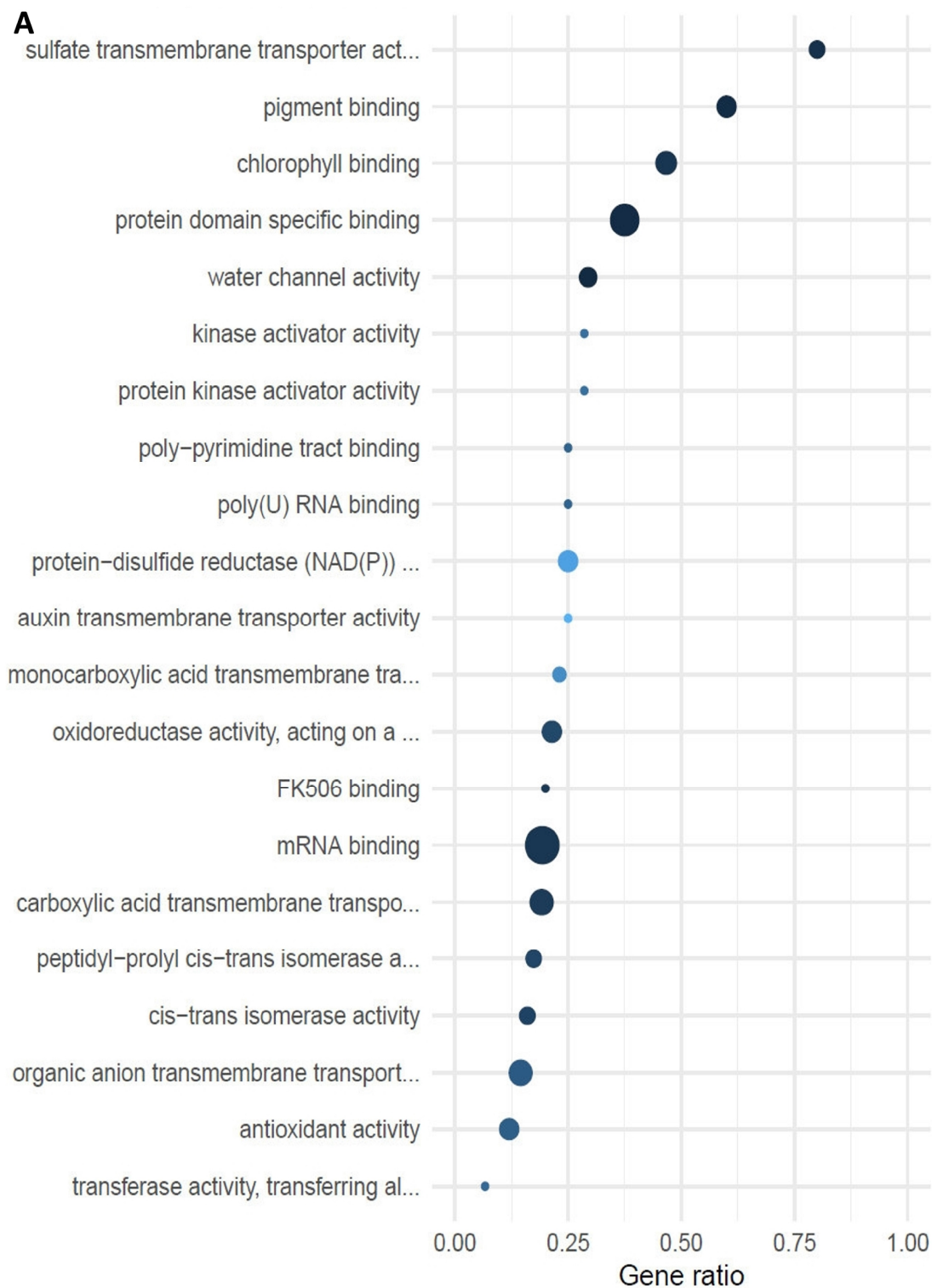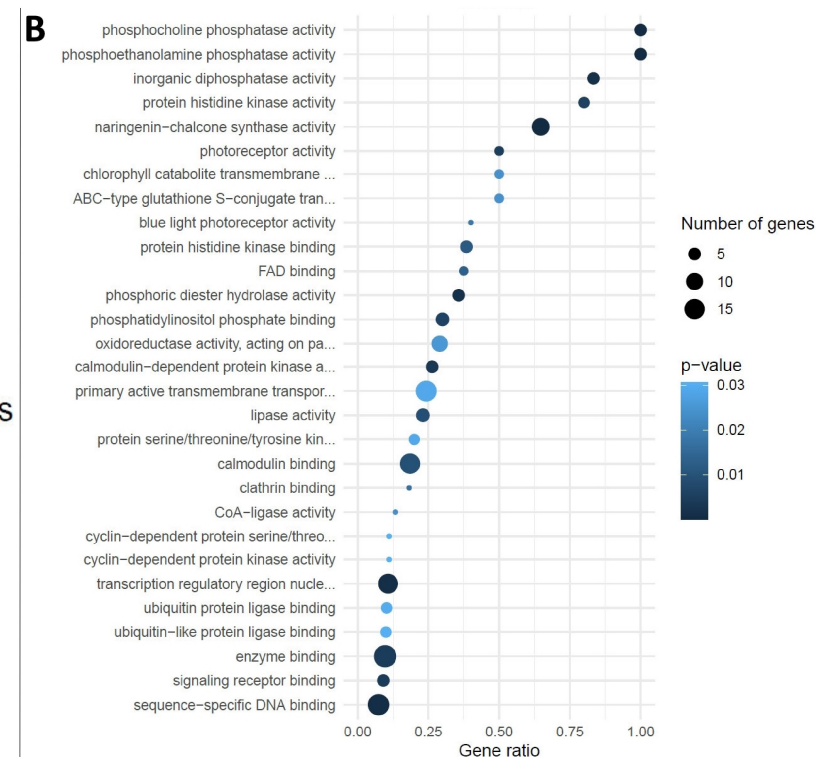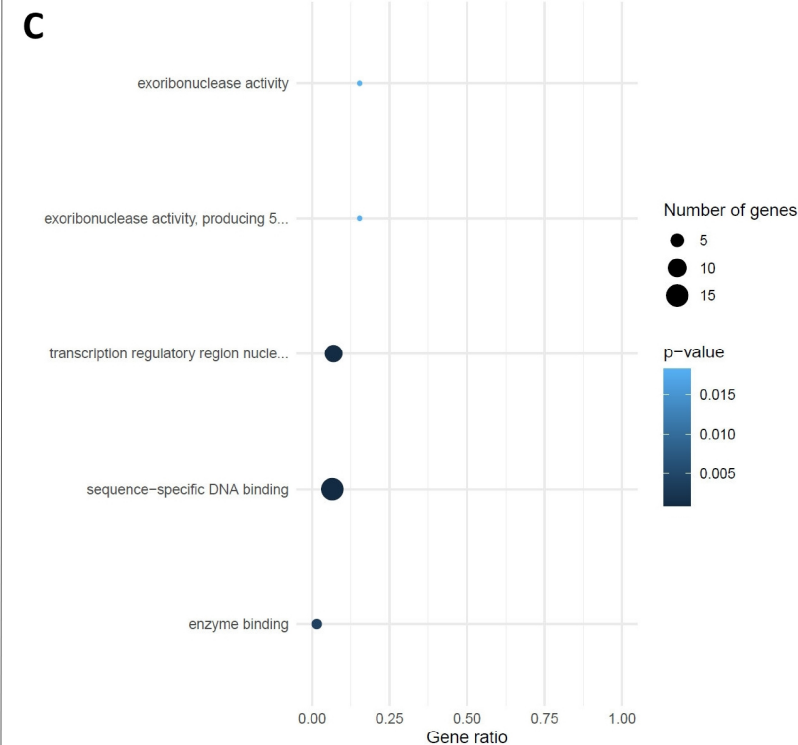

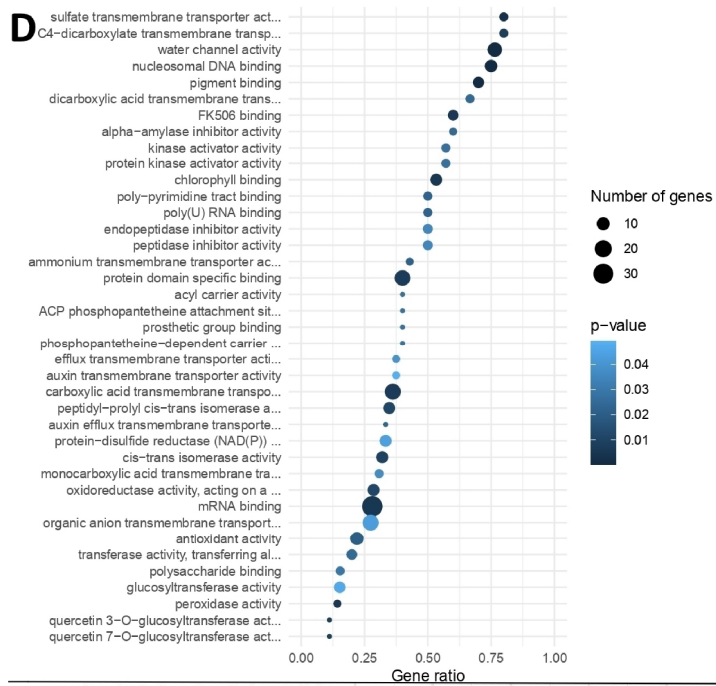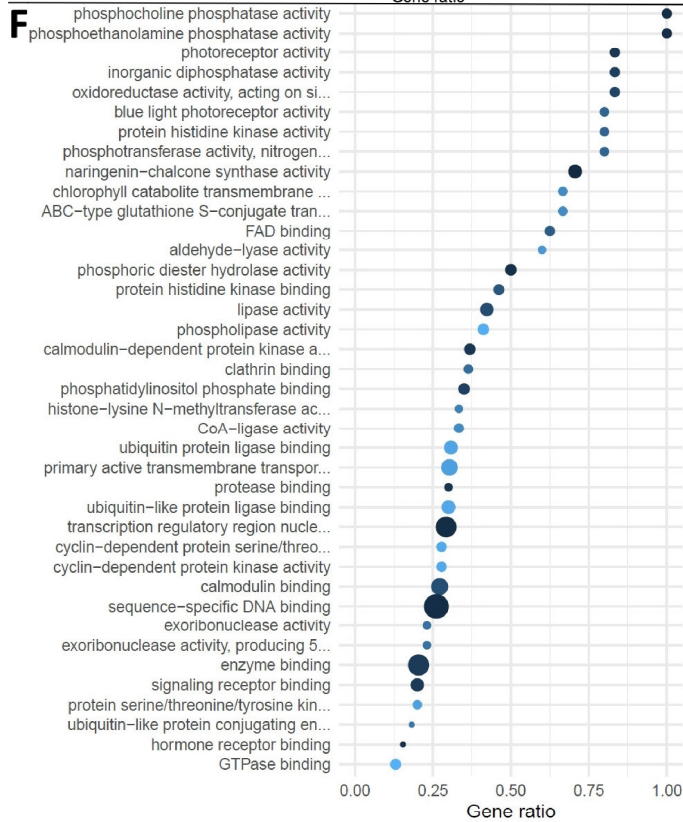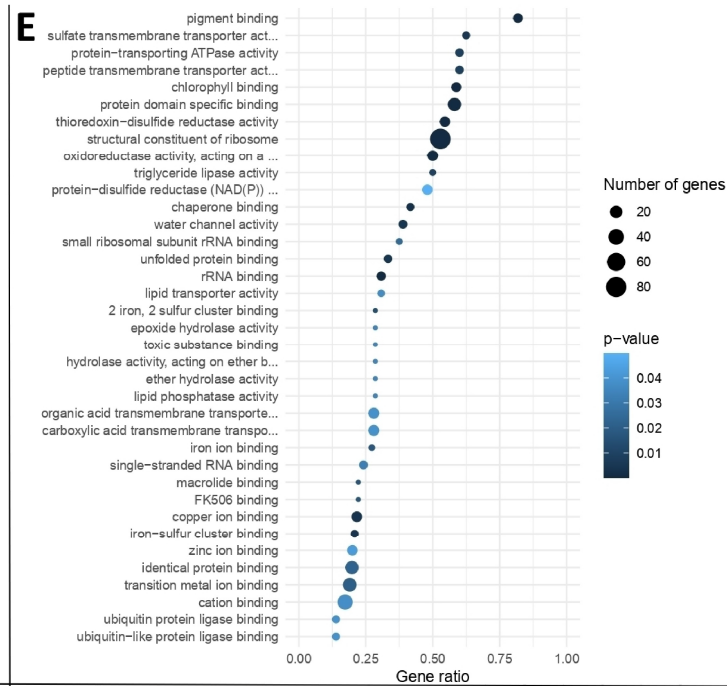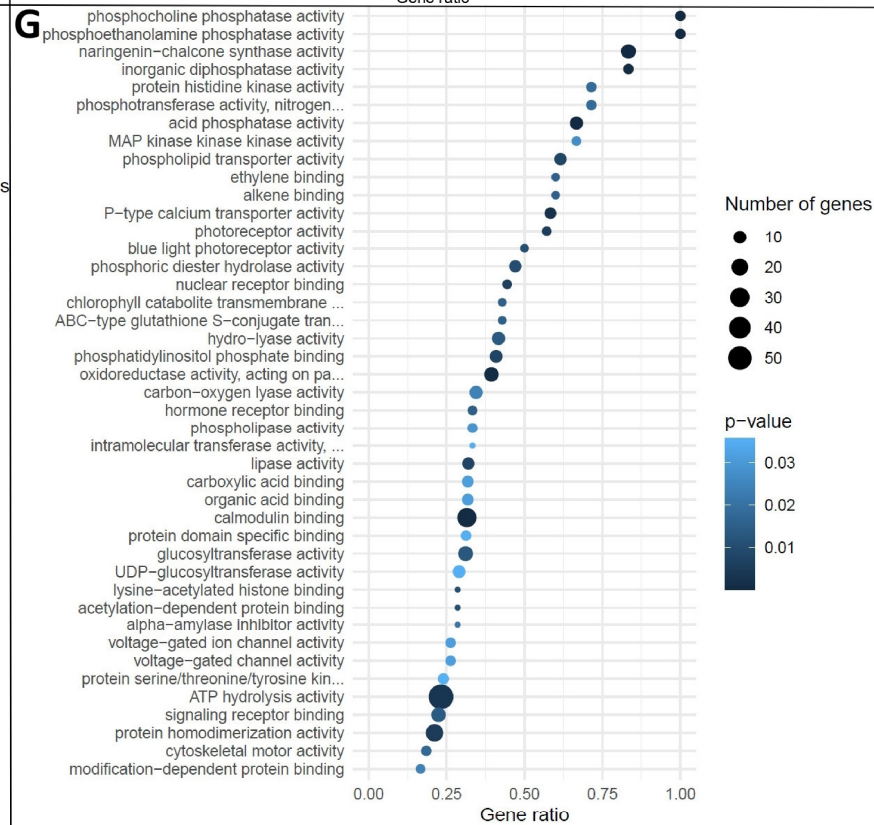

Supplement: Supplementary file 1 [file plants-12-03580-s001.zip › Figure S2.pdf]
